# Contemporary global research cultures: results from a global survey about research conditions

**DOI:** 10.12688/f1000research.171842.1

**Published:** 2026-04-19

**Authors:** Andrey Lovakov, Clemens Blümel, Stephan Stahlschmidt

**Affiliations:** 1 Research System and Science Dynamics, German Center for Higher Ed. Res. and Science Studies, Berlin, 10117, Germany

**Keywords:** research conditions, global research cultures, research diversity

## Abstract

**Background:**

The global research and higher education landscape has expanded enormously within the last 30 years. Bibliometric data reveal the increase in knowledge production, co-authorship patterns and collaborations. There are, however, only limited data that allow for comparing the research conditions among the different regions in the world that cover different disciplines and status groups. Existing surveys on research conditions are restricted to specific groups of countries, mainly in the global north.

**Method:**

Against this background, we present the results of a global survey on research conditions, conducted in partnership with the platform ResearchGate. The study draws data from more than 5,000 researchers from around the world. The study addressed key dimensions of professional environment: individual resources, perceived research conditions, work satisfaction. and network embeddedness. The survey data were enriched with other secondary data on the country level, namely, V-Dem data base as well as data on economic performance (GNI per capita).

**Results:**

The results reveal significant regional disparities in funding, infrastructure, and time for research and teaching, with academics in the Global North reporting greater satisfaction and more time for research compared to their counterparts in the Global South. Academics in autocratic regimes report satisfaction with their infrastructure, although external funding remains limited. The findings demonstrate the diversity of research conditions in the organizational settings, career incentives, and institutional conditions. These findings complement bibliometric data showing rapid publication growth in autocratic countries and a tendency towards international collaboration and highlight the diversity and complexity of global research cultures, contributing to a richer understanding of epistemic diversity.

## Introduction

The global research and higher education system has expanded enormously since the end of the Second World War ( de Solla-Price, 1963) and has continued to do so. What is more, the *participation* in knowledge production of regions beyond the global north and west has strongly increased, with China and India becoming new power houses of science ( Baker & Powell, 2024). Analysts of research production argue that an era of networked global science has emerged that is integrating global knowledge production ( Wagner, 2018). Still, however, the conditions under which knowledge is produced are different among the various world regions, given the disparities in access to economic as well as material resources and human capital ( Cantwell & Grimm, 2018; Krawczyk & Kulczycki, 2021). Moreover, the political conditions can also affect the flourishing of science ( Wagner & Jonkers, 2017) as the more recent developments in the world show. This is why in this article we strive to provide a first view on the conditions of knowledge production on a global scale.

The diversity of conditions for knowledge shows the interdependence of science with other realms of society (Weingart 2001). As various contributions on regional developments show, the organization of science in a particular region is inextricably linked with the social, economic, and political institutions existing in that region and the *longue durée* of its institutions. For instance, the legacy of soviet models of research organisation is still visible in patterns of knowledge production in post-communist countries in Eastern Europe ( Kozak et al., 2015; Lovakov et al., 2022). Consequently, local and regional institutional varieties can manifest not only in terms of impact and excellence, but also in the way national or regional systems of governance, education, and research are designed and developed. There is a large variety of how knowledge is produced, but the same goes for the societal and institutional context in which researchers are embedded.

Yet, it is difficult to tackle these varieties and to account for the diversity of research organizations globally. One of the reasons why these disparities have not been acknowledged sufficiently is because of the restriction of global comparisons using bibliometric data. Analyzing bibliometric data can be conceived as a common way of understanding the global research system (often from well-known databases such as Web of Science and/or Scopus). This approach is based on several advantages of bibliometric data: data is already being collected and there is no need to collect data. Moreover, data provides both a global view and the ability to look at the specific countries, data is well researched, and the bibliometric communities know a lot about its structure and its strengths and weaknesses ( Moed et al., 2005).

At the same time, however, these databases impose specific perspectives and render only a section of the global research community visible, respectively excluding a substantial amount of the research system in such bibliometric analysis ( Krawczyk & Kulczycki, 2021). These databases mainly cover Western, English-language journals while contributions in Chinese, Spanish, Hindu, Farsi or Russian are not well covered. It means that authors who publish in these non-indexed journals are also missing. Only published papers are included in bibliometric databases. Early career researchers without published papers are not included. Different types of scholarly output, such as monographs, edited volumes, policy-papers, reports are not represented in bibliographic databases. Similar lacks can be mounted regarding the contribution of small and medium sized publishers, who represent much of the linguistic and genre variety in the sciences ( Stephen & Stahlschmidt, 2022a). Scholars are drawing from bibliometric data bases to establish productivity of science in which scholars from the Global South and East are less represented ( Krawczyk & Kulczycki, 2021), with severe side effects. Most of the research covered in these databases originates from a small number of publishing houses located in Western centers ( Krawczyk & Kulczycki, 2021; Larivière et al., 2015). These commercial actors also control much of the current meta-data landscape in science, including citation data. Consequently, large parts of science are not represented in global science as it is visible in bibliographic databases. Contributions of specific types of knowledge, specific disciplines or fields of research, but also types of contributors are marginalized.

As a result, knowledge about the conditions and the epistemic and regional breadth of research are lacking, which would more substantially allow for exploring the relationship between (state) intervention or steering and the development of scientific research suffers from comparative perspectives including the Global South. Other sources of data on the global research system can provide very valuable complementary perspectives on bibliometrics. One of these potential data sources could be a global survey of the researchers. Such surveys may provide broader picture of the global research system and provide additional information on different conditions in which academic work. There are several examples of such surveys, where the same questions are asked to academics from different countries, e.g. “The Changing Academic Profession” ( Kehm & Teichler, 2013; Teichler et al., 2013), “The Academic Profession in the Knowledge-Based Society” ( Çalıkoğlu et al., 2023; Huang et al., 2022), “Nature Post-Doctoral Survey 2023” ( Nordling, 2023), “2020 Nature Postdoc survey” ( Woolston, 2020), DataON survey on data provision, use or even reuse ( Tenopir et al., 2015). However, these surveys either cover a limited number of countries (again, mostly from the Global North) or focus on the specific group of academics. We still don’t know much about what researchers beyond the Global North think about their work and how they perceive the conditions of their scientific activities. Since surveys and studies about researchers’ perceptions focus predominately on Europe, North America and Asia, little is known about in the countries of the Global South.

In order to get a complementary view on the diversity of research carried out and the financial, institutional, and political conditions under which this is done, the German Centre for Higher Education Research and Science Studies (DZHW) partnering with the platform ResearchGate conducted the State of the Research Community survey, aiming to cover respondents from countries of the global center and periphery of science. This survey stands out from other surveys due to several key differences in its approach and methodology. Unlike many other surveys that focus on specific regions or countries, this survey includes responses from researchers at all stages around the world. This global scope allows for a broader and more diverse understanding of the state of the research community, capturing both global trends and the specificities of different groups of countries. The unique aspect of this survey is the use of ResearchGate as the data collection platform. Researchers self-select onto this platform with no barriers to entry and are verified by their email address, ensuring that respondents are genuine members of the research community. This contrasts with other surveys, which may use random sampling or target specific institutions. The platform is widely used by researchers around the world, giving the survey a wide reach and a diverse respondent base. The survey is not based on bibliometric sampling, which relies on publication data and can introduce biases and limitations, such as over-representation of highly published researchers and exclusion of early-career researchers or those in less well-known institutions. The survey eliminates these biases, ensuring a more inclusive and diverse sample of the research community.

While the survey provides valuable insights into the conditions under which academics work, it is also important to acknowledge their own limitations. A major limitation is the lack of data on sensitive questions, as some respondents chose not to answer certain questions that they found problematic. This non-response may lead to an incomplete understanding of critical issues. Additionally, language bias may affect the results, as the survey may not fully capture the perspectives of non-native English speakers. Furthermore, the use of the ResearchGate platform for data collection introduces platform bias, as the sample primarily includes researchers who are active on ResearchGate, potentially excluding those who use other platforms or are less digitally engaged. These limitations should be considered when interpreting the results.

## Dimensions of the global research system

Countries vary in many dimensions. However, available resources and political regime may be named as the key factors affecting both knowledge production and conditions in which this knowledge is produced. Countries’ economic power is strictly related to its research production and work conditions for academics ( King, 2004; May, 1997). Economically powerful countries typically have greater financial resources to invest in R&D, leading to higher research production. These investments manifest in well-funded universities, advanced laboratories, and access to cutting-edge technology, which collectively create better environment for high-quality research. Moreover, in economically powerful countries, academics often enjoy better work conditions, including higher salaries, better resources available, and more opportunities for professional development. These favorable conditions attract and retain top talent, further enhancing the country’s research output. On the contrary, countries with weaker economies may struggle to provide adequate funding and resources for R&D, resulting in less prolific research production and less favorable working conditions for academics. This can lead to brain drain, where highly skilled researchers migrate to countries with better economic conditions and research infrastructure ( Sanliturk et al., 2023). Thus, economic power plays a critical role in shaping research landscape and the work environment for academics.

The political regime of a country may also significantly influence its research production and the work conditions for academics. In the 1930s, Robert K. Merton dealt with the question in which societal context the sciences can flourish best. He argued that science favors a democratic order which would allow for the institutionalized norms of science of universalism and organized skepticism to function. Whenever these norms are not guaranteed by state or society, science institutions are harmed ( Merton, 1973). Today, however, many authoritarian regimes are successful in research, and it is argued that the thesis of Merton cannot easily be upheld. Democratic western countries remained large producers of scholarly knowledge for decades, with countries such as the US, the UK, France, Germany, Canada. Yet, based on publication numbers derived from bibliographic databases, authoritarian regimes show high numbers of growth. Marginson (2022) shows that the annual growth rate of papers from countries considered authoritarian such as Iran (21%), Pakistan (15%) or China (14%) is higher than for most of the countries with a democratic order (measured based on Scopus). The share of China in global publications has risen from 1.6% in 1995 to 23.1% in 2020. It thereby has surpassed the US as the largest producer of output as represented in scientific publications. During the same period the share of the US has decreased from 32.5% to 17.1%, while Germany’s share fell from 6.5 to 4%. There is also a strong increase of China in the realm of highly cited literature in the same period, which is often associated with academic impact, visibility, or at times even, quality ( Stephen & Stahlschmidt, 2022b). On the other hand, Kinzelbach et al. (2022) argued that for individual universities data suggest that higher scores in Academic Freedom Index correlate with higher ranking positions. These findings indicate that there may be no simple relationship between national democratic order and the flourishing of science. There are attempts to explain or at least provide interpretations for the success and rising participation of countries with authoritarian regimes. Marginson (2022) provides one approach by emphasizing that considering nations as the silos for science production may be somewhat outdated given the increased global collaboration of scholars, particularly in specific fields. Global science in his view is a specific mode of knowledge production. He noted that “… the global system is distinct and different, not just existing separately from national science systems but also orthogonal to them” ( Marginson, 2022, p. 1567).

To capture the research conditions in which academics work, we examine three key dimensions of their professional environment: individual resources, perceived research conditions, and network embeddedness. The first dimension includes tangible elements such as the distribution of time between research and teaching, availability of research funding, and access to adequate facilities and equipment as these factors are crucial for conducting high-quality research. The second dimension captures subjective evaluations, such as perceived academic freedom and the perceived ability to contribute meaningfully to science. These perceptions often mediate how structural conditions are experienced. The third dimension concerns the academic’s integration into scholarly networks, including participation in the international peer community and the structure of the academic labor market, which affects job security, mobility, and access to collaboration. Together, these aspects offer a general understanding of the conditions under which academics work.

## Data and methods

### Questionnaire

The questionnaire includes a set of questions covering the key aspects of professional environment discussed above.

***Share of time spent on research and teaching***

Share of time spent on research and teaching were measured by question “In the last twelve months, what percentage of your time at work did you spend on the following duties? All responses need to add up to 100: Research (carrying out or overseeing research, supervising (doctoral) students, finding & reading papers), Applying for funding (writing/co-writing grants), Publishing (writing & co-writing, peer-review, data storage, sharing), Teaching, Administrative duties, Other.

***Satisfaction with the funding possibilities and research facilities and equipment***

Satisfaction with the funding possibilities and research facilities and equipment for research was measured by three questions: “In the last 12 months, how satisfied have you been with the available research facilities and equipment for your research work?”; “In the last 12 months, how satisfied have you been with the funding for research projects available at your institution (excluding third−party funding)?”, “In the last 12 months, how satisfied have you been with the available third−party funding opportunities?”. Respondents used 5-point Likert scale to answer these questions: (1) Very dissatisfied, (2) Dissatisfied, (3) Neutral, (4) Satisfied, (5) Very satisfied.

***Perceived academic freedom***

We used the item battery of the Academic Freedom Index (AFI) ( Spannagel & Kinzelbach, 2023) in order to operationalize the concept of academic freedom, which has two dimensions. The first question refers to freedom for topical choice in research and teaching “To what extent are you free to develop and pursue your own research and teaching agendas without interference by non-academic actors”. Note that non-interference does not exclusively relate to the state as one of the most relevant actors, but may also include commercial actors, or religious groups, such as the church. The second question refers to freedom for getting funding for research “To what extent are you free to pursue your personal research interests with the funding you have available?”. Respondents used 5-point Likert scale to answer these questions: (1) Completely restricted, (2) Severely restricted, (3) Moderately restricted, (4) Mostly free, (5) Fully free.

***Structure of the academic labor market in terms of representation of different roles and types of contracts***

In order to characterize the structure of the academic labor market, questions were asked about the current primary role/position (e.g. full professor, associate professor, postdoctoral fellow), employment status (e.g. employed full-time on a permanent contract, employed full-time on a temporary contract), and the next career milestone (e.g. tenure, a promotion, leading own research group or lab, publication in a highly reputable journal).

***Importance of the international peer community***

The importance of the international peer community was measured by the question “How international or local is the professional peer community that you want to advance your career in?” Respondents used 5-point Likert scale to answer this question: (1) Exclusively national/local, (2) More national/local, (3) Balanced, (4) Somewhat international, (5) Highly international.

***Perceived ability to contribute to science***

Perceived ability to contribute to science and also to other aspects of work and family were measured by three questions: “Looking back over the last 12 months, do you feel like you spent too much, too little, or the right amount of time on the following areas?” Areas included “My ability to contribute to science”, “My private/family life”, and “Other aspects of my work, as administrative duties or teaching”. Respondents used 3-point Likert scale to answer these questions: (1) Too little, (2) About right, (3) Too much.

### Data collection

The *State of the Research Community* survey results from a cooperation between the German Centre for Higher Education Research and Science Studies (DZHW) and ResearchGate and was conducted from April to July 2022. The survey is based on a jointly developed questionnaire with 51 questions on the topics career prospects, current research environment, participation in scientific discourse, work satisfaction and demographic covariates. ResearchGate implemented the survey via the third-party software SurveyMonkey. Participation was facilitated via a single URL to the online questionnaire for all participants to safeguard the anonymity of the answers. This link was shared in a post on the ResearchGate blog ^1^ and the post was prominently mentioned in a simultaneous press release by DZHW ^2^. The openly published link allowed also scientists without a ResearchGate account to participate. In addition, a subset of ResearchGate users were made aware of the survey via a pop-up window while being logged into their ResearchGate account. This special access to respondents allowed ResearchGate to manage the quantity of respondents. Upon reaching the goal of 10 k respondents the link to the survey form was deactivated. ResearchGate checked the open text fields on the potential presence of any personal information before passing the data on to DZHW. An initial analysis on the demographic characteristics of the respondents including the calculation of the completion rate of 60% has been published on the ResearchGate blog ^3^.

### Additional data

The survey data were enriched with other country-level data. First, in order to establish an economic perspective on research conditions, the data was enriched with income data from the World Bank. Based on GNI per capita, countries were divided into four groups: low-income economies, lower-middle-income economies, upper-middle-income economies, high-income economies. Second, the data was also enriched with the data on the political regime. Based on the data from the V-Dem dataset (version 13, Coppedge et al., 2023) from the V-Dem Institute at the University of Gothenburg, the countries were divided into four regimes ( *v2x_regime*): Closed autocracy, Electoral autocracy, Electoral democracy, Liberal democracy.

### Analytical strategy

The analytical strategy was as follows. The dependent variables were numeric answers to the questions about main aspects of their professional environment. The main independent variables – regime and income – were transformed into *n* – 1 dummy variables (0 or 1). This allows the use of them in regression models. If the coefficient on the regime or income dummy is different from zero, there are differences between two regimes or income groups. Two control variables are also included in the models: gender (male, female, other), position (Full professor, Assistant/Associate Professor, PhD candidate, Postdoctoral fellow, other). The survey data are nested: individual respondents are nested within countries. This means that individual responses are not independent of each other. The linear mixed effects model was used to account for this non-independence. The linear effects mixed model (estimated using REML and the nloptwrap optimizer) was fitted by *lme4* ( Bates et al., 2015). Each country can have its own interception in the model.

## Results

### Broad participation

**Figure 1.**
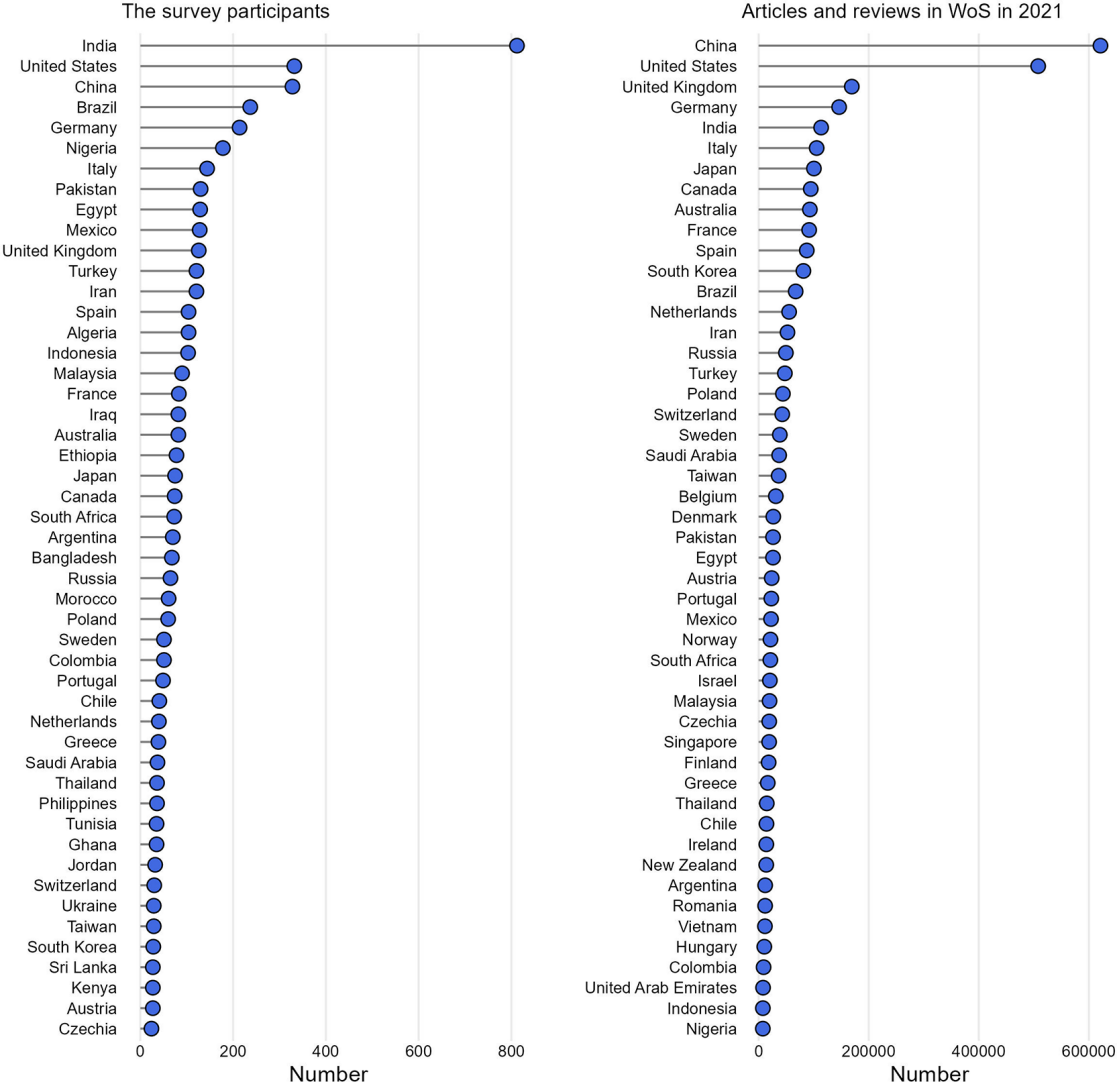
Top countries by number of survey participants (left panel) and by number of articles and reviews in WoS in 2021 (right panel).

Over 8,000 researchers from different disciplines and at different stages of their careers, working in countries all over the world, took part in the survey. However, only 5,597 of them indicated the country in which they were working at the time of the survey. The survey reached the broad academic community. An analysis of the distribution of participants by country shows that researchers from the Global South are more strongly represented among the respondents of the survey, compared to bibliometric retrieved from Web of Science (WoS) database in 2021. The rankings of countries based on the share of participants and based on the share of articles and reviews in Web of Science (WoS) are different (see Figure 1). Data about the location of the respondents shows the broad coverage of regions in the survey. However, the data is also biased, as respondents from Western Europe make a larger share than actual share of world population. This may also be related to regional and cultural proximity of ResearchGate to Europeans, since ResearchGate is located in Western Europe. In addition, the proportion of Chinese researchers is much smaller than China’s share on world publications as represented by WoS. Nevertheless, the participation of respondents, as regards the physical location of the respondents, is much more diverse than science-related surveys on other subjects which often focus on very few countries outside the Global West.

**Figure 2.**
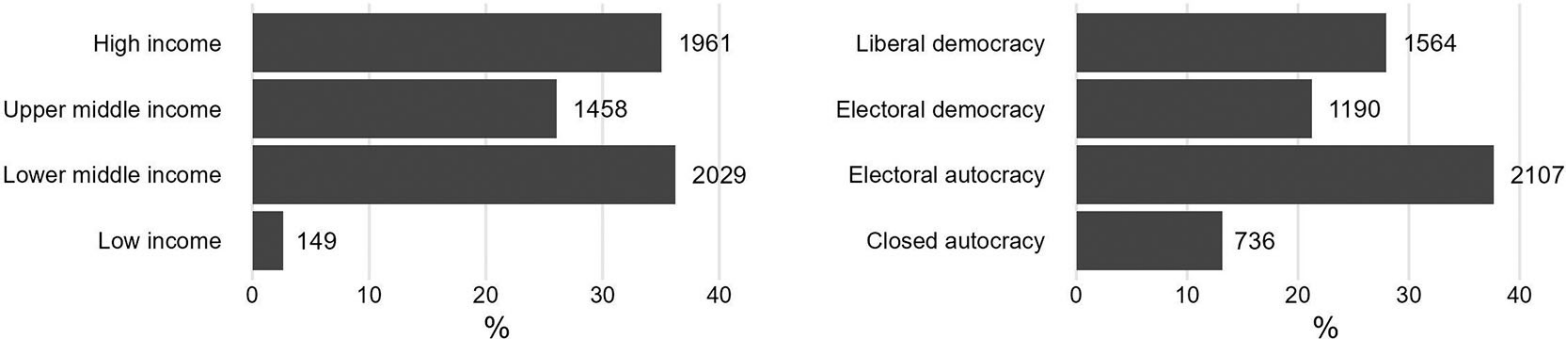
XXX.

Figure 2 shows the distribution of the participants by income and political regime groups. Participants are distributed by groups relatively equal, except low-income group with that are affiliated only 149 participants. To make income groups more comparable we merged low-income and lower-middle-income groups into one group for further analysis. Distribution of respondents by groups of countries with different income levels and political regimes are shown in Appendix (Figures A1 and A2).

### Differences between country groups in the share of time spent on research

**Figure 3.**
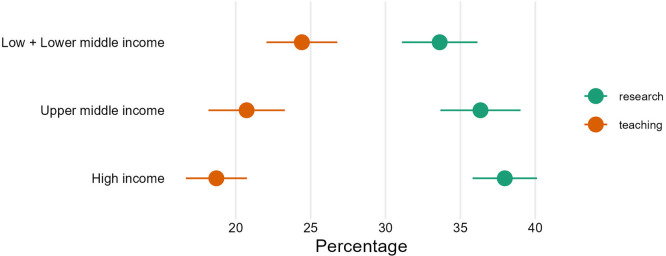
Estimated marginal means and 95% confidence intervals for time spent on research and teaching in countries with different income level.

Research is the most common type of duties in the sample. On average, participants spent 36% of their time on research. Teaching follows research at around 22%. However, there are significant differences between groups of countries in the proportion of time spent on research and teaching (see results of regression analysis in Table A1 in Appendix). The higher income, the higher share of time spent on research and lower share of time spent on teaching (see Figure 3). Participants from low- and lower-middle-income countries report the lowest proportion of time spent on research (34%, 95% CI [31%; 36%]) and the highest proportion of time spent on teaching (24%, 95% CI [22%; 27%]). Participants from high-income countries report the highest proportion of time spent on research (38%, 95% CI [36%; 40%]) and the lowest proportion of time spent on teaching (19%, 95% CI [17%; 21%]). Higher teaching duties can be considered as a source of inequality in the resources available for research. These time resources appear to be a product of economic scarcity and economic considerations regarding the priorities of scientific personnel. Consequently, this inequality in resources, including time resources, may lead to the differences in research output and impact that are consistently reported in studies based on bibliometric databases.

### Differences between groups of countries in satisfaction of the funding and research facilities

**Figure 4.**
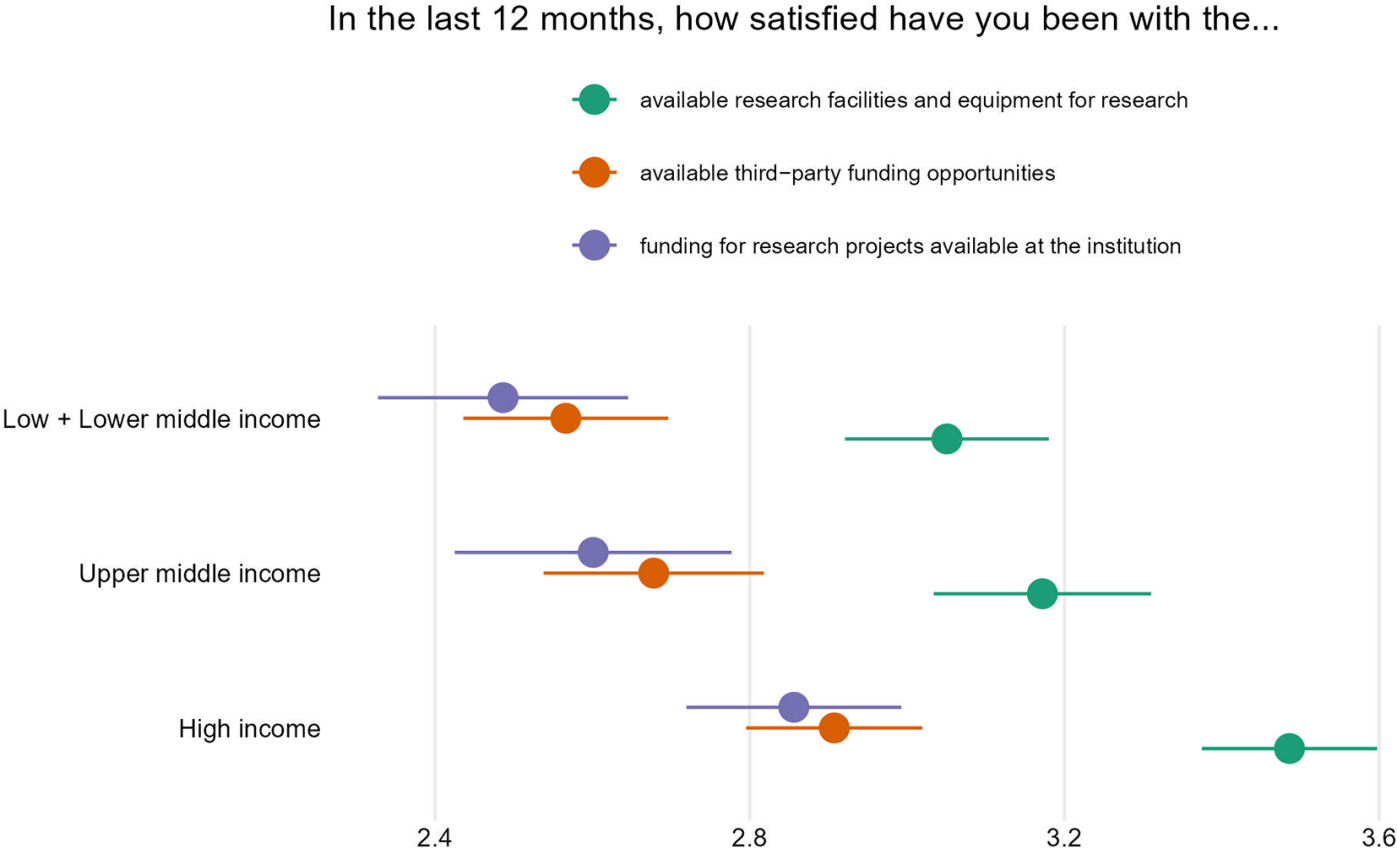
Estimated marginal means and 95% confidence intervals for satisfaction of the funding and research facilities and equipment in countries with different income level.

The technical and financial resources provided for research are among the most relevant conditions. Particularly in several of the most competitive subjects such as bioengineering, material science or semiconductors, there is a need for particularly expensive technical infrastructures, such as laboratory equipment, machinery, or clean rooms. These technical infrastructures not only require financial resources, but also availability of components, some of which are issued or even sanctioned. But technical infrastructures also require trained personnel who can maintain, curate, and use the infrastructure and machines efficiently. Against this background, the differences in technical infrastructure are particularly striking. On average, respondents from low-income countries have less access to technical facilities and should therefore report less satisfaction with technical facilities. Our results show the differences in satisfaction with funding and research facilities and equipment between countries groups (see results of regression analysis in Table A2 in Appendix). Participants from high-income countries report significantly higher level of satisfaction with the available research facilities and equipment (3.49, 95% CI [3.37; 3.60]), the funding for research projects available at the institution (2.86, 95% CI [2.72; 2.99]), and the available third-party funding opportunities (2.91, 95% CI [2.79; 3.02]) compared to researchers from upper-middle-income (3.17, 95% CI [3.03; 3.31], 2.60, 95% CI [2.42; 2.78], and 2.68, 95% CI [2.54; 2.82], respectively) and low- + lower-middle-income countries (3.05, 95% CI [2.92; 3.18], 2.49, 95% CI [2.33; 2.65], and 2.57, 95% CI [2.43; 2.70], respectively). The difference in these measures between upper-middle-income and low- + lower-middle-income countries are not so pronounced. However, in general, the lower income, the lower satisfaction (see Figure 4). The differences here provide rather clear indication of different material conditions for the conduct of research within different global regions. Another visible pattern is that in general researchers in all countries are less satisfied with funding (means are vary between 2.49, 95% CI [2.33; 2.65]) and 2.91, 95% CI [2.79; 3.02]) than with research facilities and equipment (means are vary between 3.05, 95% CI [2.92; 3.18] and 3.49, 95% CI [3.37; 3.60]). These results suggest that researchers in all countries suffer from nonperfect funding systems. However, researchers from high-income countries on average suffer less compared to researchers from the rest of the world.

### Differences between country groups in perceived academic freedom

**Figure 5.**
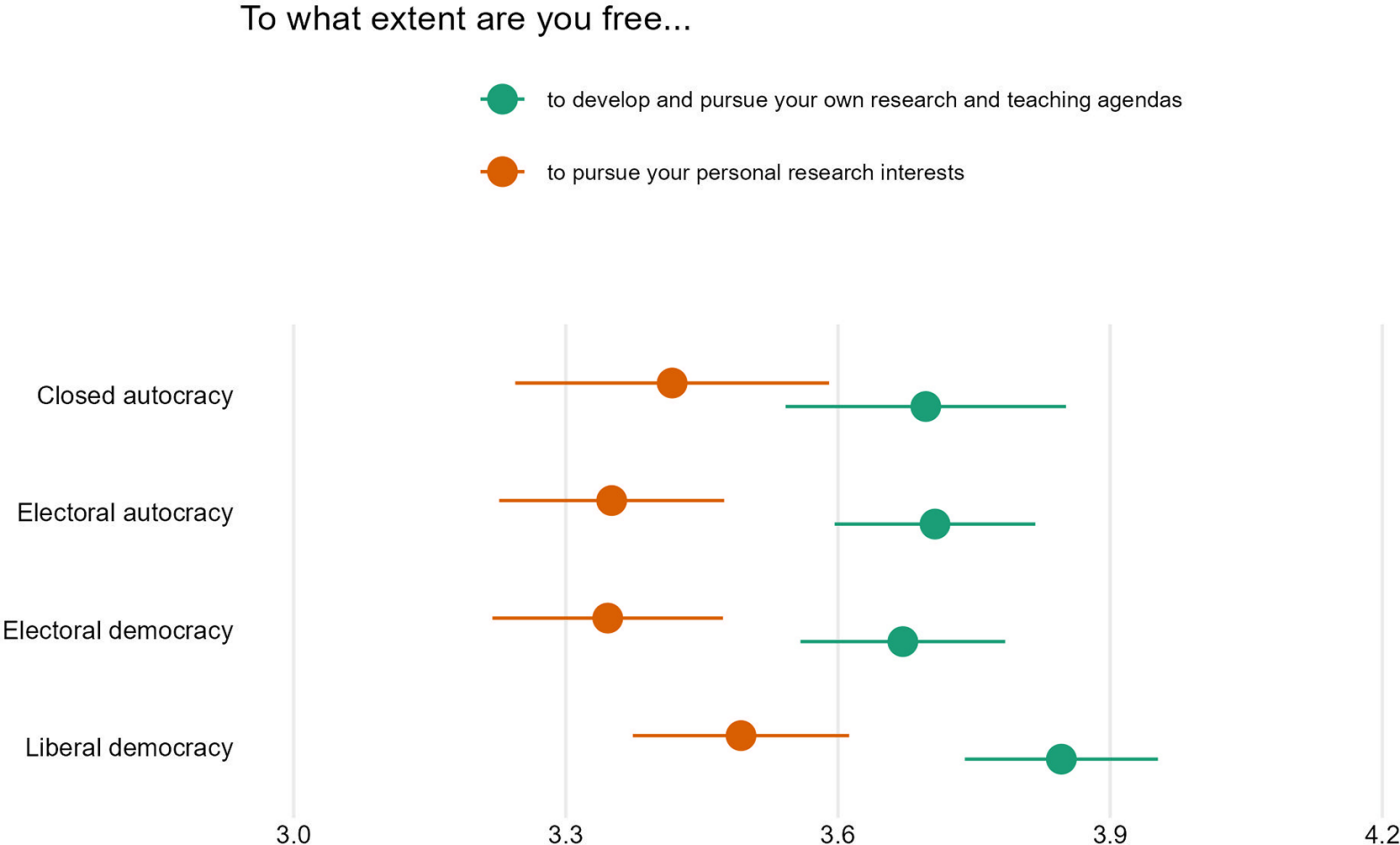
Estimated marginal means and 95% confidence intervals for perceived academic freedom in countries with different political regimes.

By enriching our survey with additional data on political regimes, we were also able to compare the perceived freedom to pursue research agendas in relation to the different political regimes. The results show the differences in perceived academic freedom between groups of countries (see results of regression analysis in Table A3 in Appendix). However, the differences are less pronounced than might be expected, especially when compared with the data from ( Kinzelbach et al., 2022; Spannagel & Kinzelbach, 2023). Only respondents from countries in the liberal democracy group report a slightly higher level of perceived freedom both to develop and pursue their own research and teaching agendas without interference from non-academic actors (3.85, 95% CI [3.74; 3.95]) and to pursue personal research interests with the available funding (3.49, 95% CI [3.37; 3.61]) compared to respondents from the electoral democracy (3.67, 95% CI [3.56; 3.79] and 3.35, 95% CI [3.22; 3.48], respectively) and electoral autocracy (3.71, 95% CI [3.59; 3.82] and 3.35, 95% CI [3.22; 3.48], respectively) groups (see Figure 5). However, respondents from countries with a closed autocratic regime are not statistically different from the other groups. The means in this group have wider confidence intervals, which may be due to a higher dispersion of the responses in this group. This dispersion may be an indicator of the greater inequality of working conditions in closed autocracies, where one group of researchers is given far more resources and freedom than others. Another possible explanation for the lack of substantial difference between democratic and autocratic regimes may be the differences in the respondents’ internal definition of academic freedom, and also in the perceived standards that researchers in different countries and academic systems may have. Future research could more systematically ask for specific conceptions and frameworks of academic freedom research.

### Differences in perception of wider working conditions

**Figure 6.**
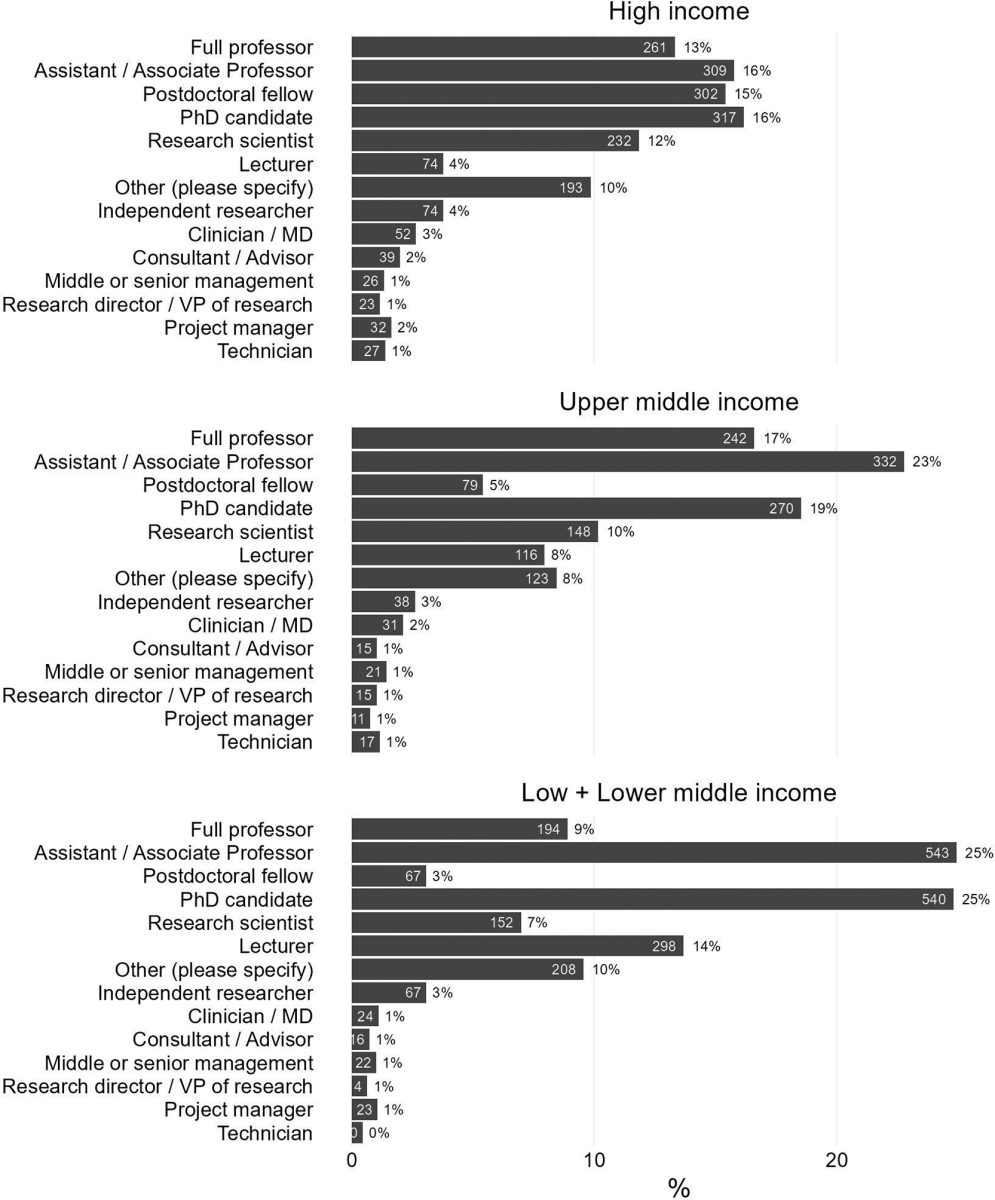
Proportions of different roles/positions among respondents from countries with different income level.

Differences between the countries become more profound when taking other aspects of working conditions into account. As regards the representation of different academic groups in the survey, it is striking that the share of postdoctoral fellows is significantly lower among respondents from the Global South (see Figure 6). If the income perspective is taken, about 15% of respondents from high-income countries describe themselves as postdoctoral fellows, while only 5% fall in that category in upper-middle-income countries or 3% in low- or lower-middle-income countries. One possible explanation is that postdocs in western high-income countries feel stronger inclined to be present on academic social networks in order to disseminate their work or to present themselves. The most common categories among the respondents from the Global South countries are Assistant/Associate professors, PhD candidates, and Lecturers. Yet, the strong presence of postdocs among the respondents in the West seems to generate a range of structural differences in the survey as we will see.

**Figure 7.**
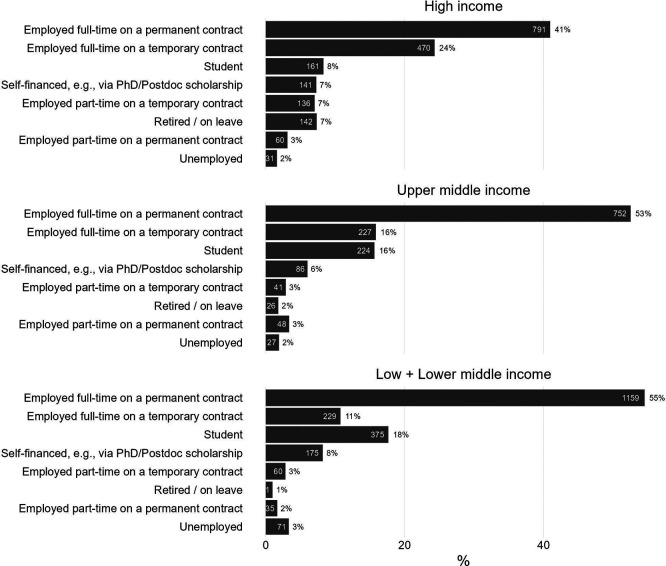
Proportions of different employment statuses among respondents from countries with different income level.

We also find that a larger share of the researchers in the Global North is on fixed contracts (24% in high-income countries vs 16% and 11% in other groups), whereas the share of respondents on tenured or long-term positions is significantly lower compared to respondents from other world regions (41% in high-income countries vs 53% and 55% in other groups) (see Figure 7).

**Figure 8.**
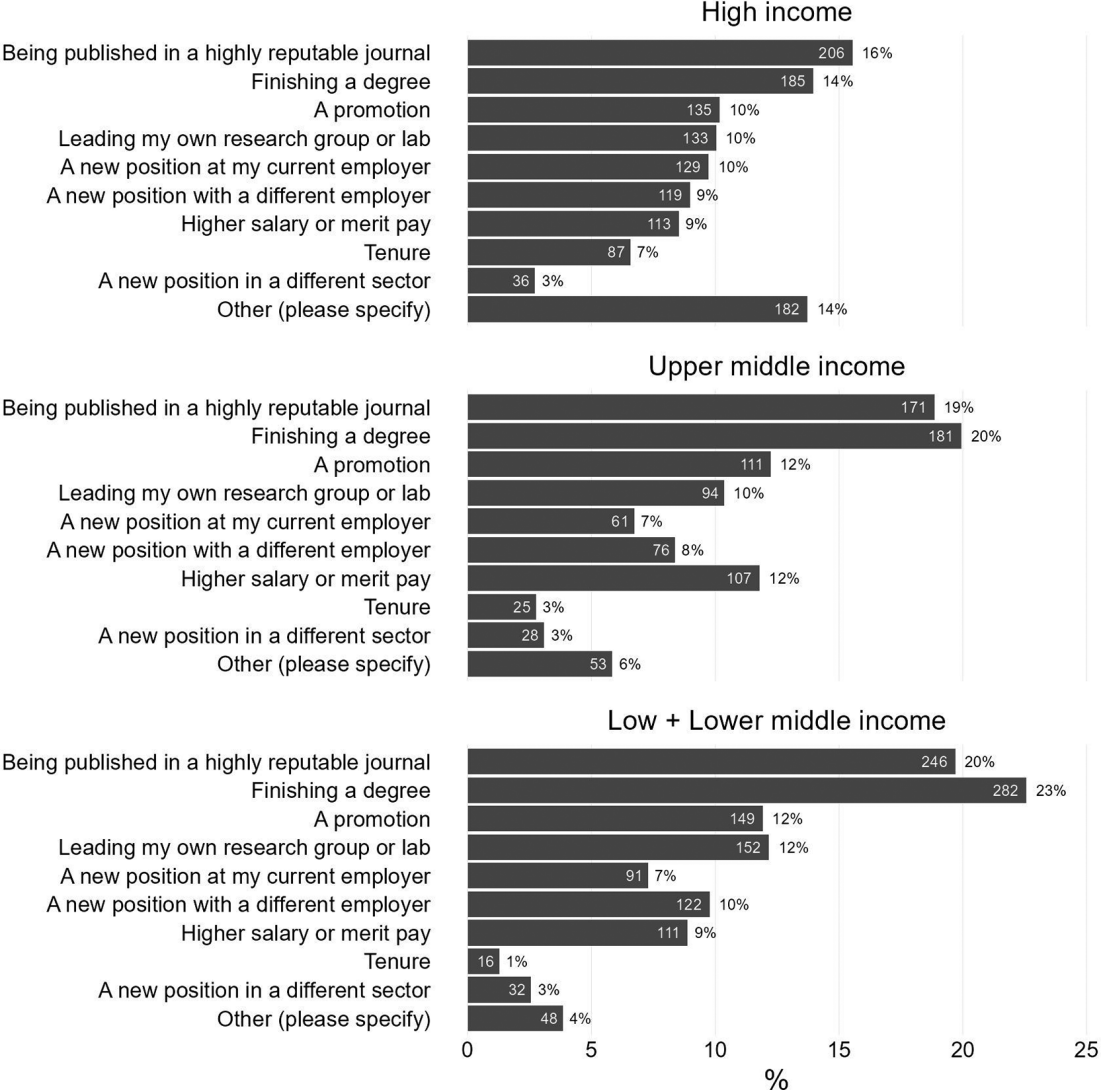
Proportions of next career milestones among respondents from countries with different income level.

**Figure 9.**
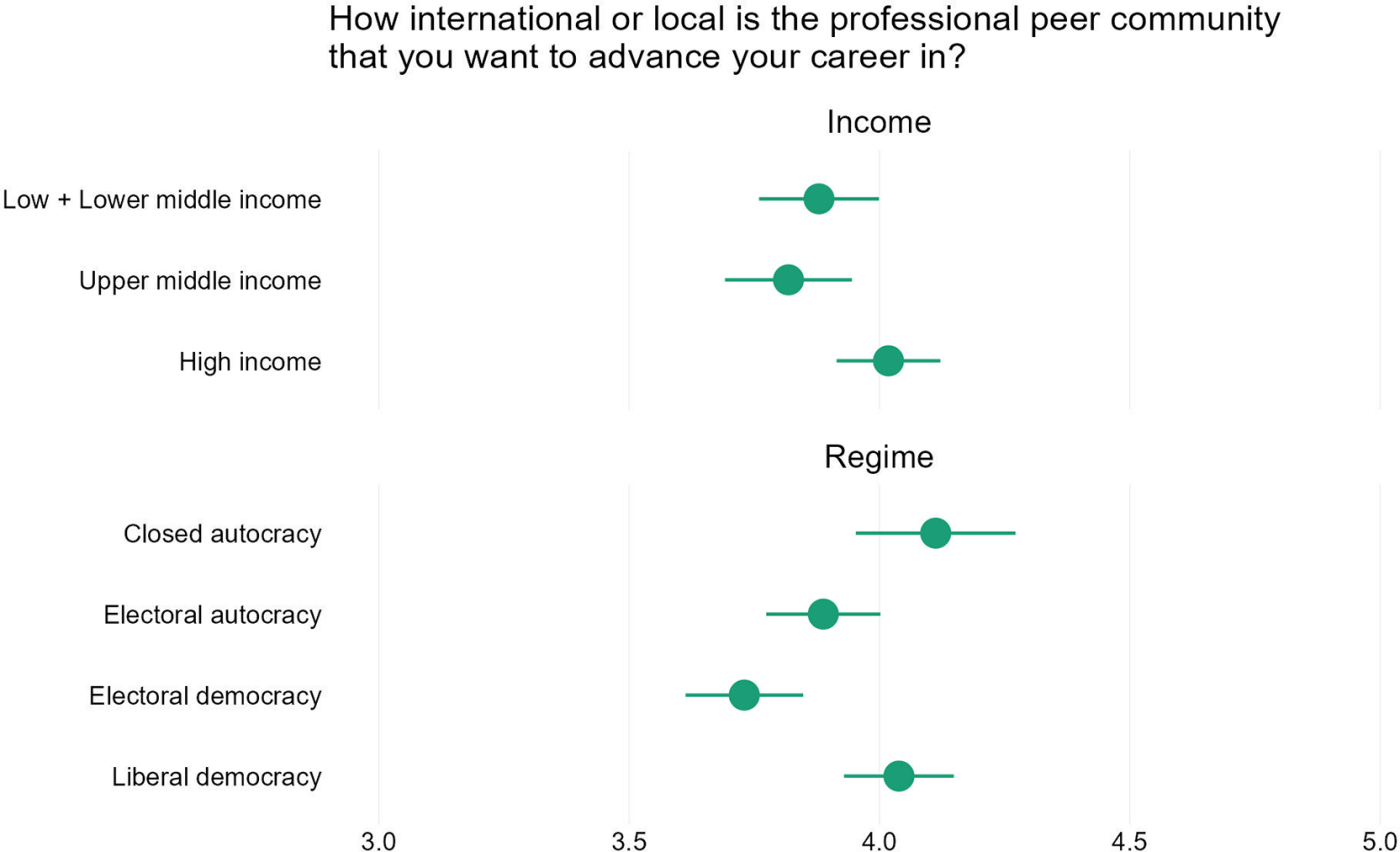
Estimated marginal means and 95% confidence intervals for importance of the international peer community in countries with different income and political regimes.

Analysis of the next career milestones shows that respondents from all country groups have similar plans (see Figure 8). The most common milestones include publication in a highly reputable journal, finishing degree, and promotion. An interesting aspect is as to whether national or international networks or resources are considered important to achieve this goal or to reach the next career step. Here, we find that respondents from all country groups report a high importance of the international professional peer community (see Figure 9). The data seem to suggest that relying solely on national resources and networks only does not seem to be a viable option. There are also differences in importance of international community between regions and between the different regimes (see Table A4 in Appendix). International community is more important not only for researchers from high-income liberal democracies (4.02, 95% CI [3.91; 4.12]) but also for researchers from closed autocracies (4.11, 95% CI [3.95; 4.28]). The higher importance of the international community for researchers from closed autocracies may be due to crucial restrictions in national academic system.

**Figure 10.**
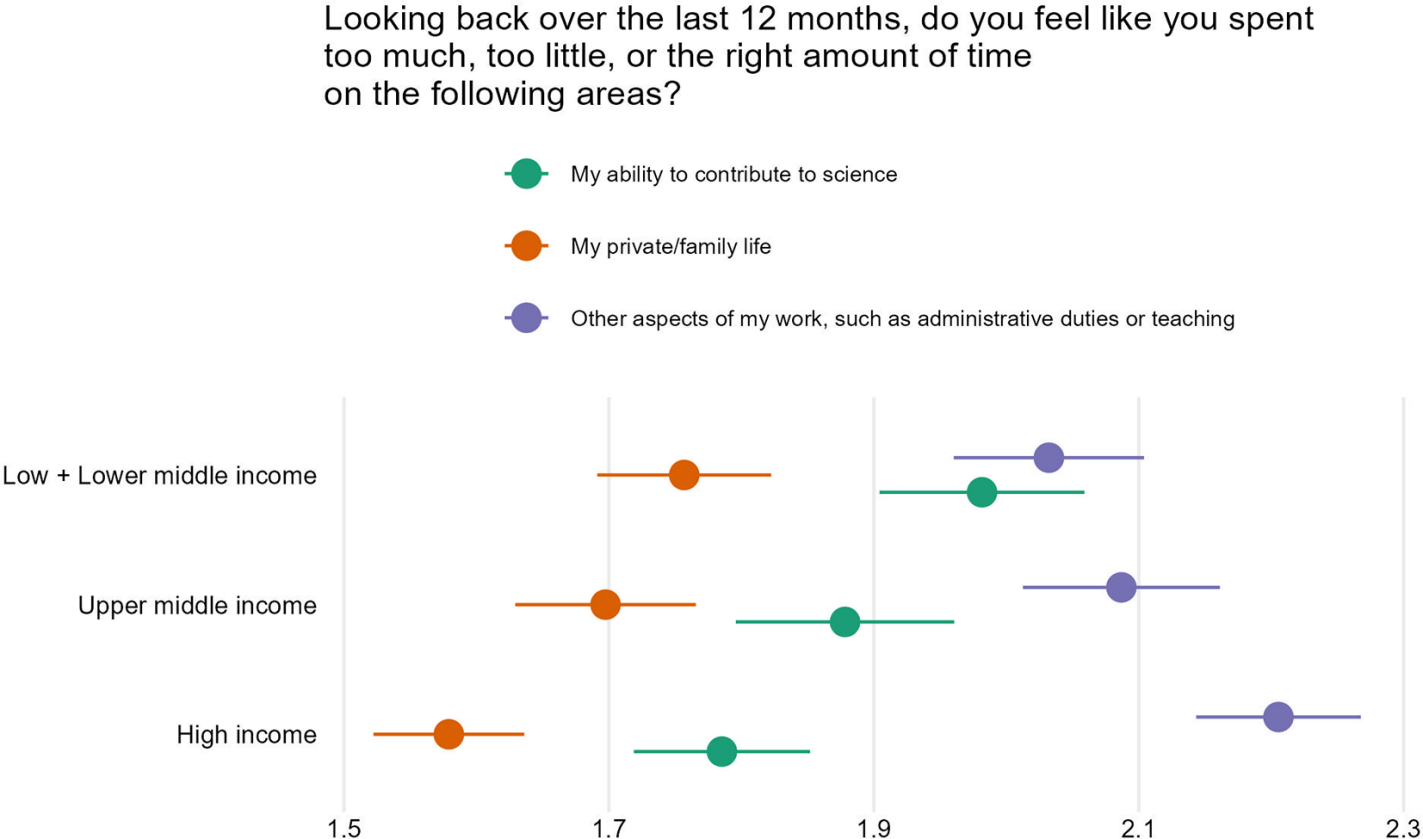
Estimated marginal means and 95% confidence intervals for satisfaction with contribution to science and private life.

Researchers from the Global West appear to be under strong pressure, with apparently high expectations regarding the scientific relevance of the use of their research. When asked to assess their contributions to science, respondents from less economically developed countries report these observations to a lesser extent (see Table A5 in the Appendix). The mean for high-income group is 1.79, 95% CI [1.72; 1.85], the mean for the upper-middle-income group is 1.88, 95% CI [1.79; 1.96], and the mean for the low- and lower-middle-income group is 1.98, 95% CI [1.90; 2.06] (see Figure 10). Furthermore, the perception of low contribution in Western academia is not related to the effort put into academic work. There are more respondents from the Global West who report that their efforts to contribute to science would be at the expense of their private life (means are 1.58, 95% CI [1.52; 1.64] vs 1.70, 95% CI [1.63; 1.77] and 1.76, 95% CI [1.69; 1.82]). Respondents from the high-income group report that they contribute too much to other work-related tasks apart from science, such as administration. Whereas respondent from the upper-middle-income and low- and lower-middle-income groups have similar perceptions of their contribution to other work-related tasks. The mean for the high-income group is 2.21, 95% CI [2.14; 2.27], the mean for the upper-middle-income group is 2.09, 95% CI [2.01; 2.16], and the mean for the low- and lower-middle-income group is 2.03, 95% CI [1.96; 2.11]. Summarizing, respondents from the Global South have more balanced responses, with individual expectations and practice more closely aligned than respondents from the Global North. The responses show that the competitive culture and fixed-term contracts in the Global North have a more fundamental impact on perceptions of freedom to pursue research on a long-term basis.

## Conclusion

The debate on global conditions for science also reveals the current structures of modern scientific infrastructures including the publishing system as well as its information infrastructures, which renders part of the research produced invisible. Such misrepresentation has adverse effects for the mis-location of academic conduct or misconduct and produces logic of center and periphery ( Krawczyk and Kulczycki 2021). There is a growing need to acknowledge and reflect existing injustice and center-periphery structures in the science system. Such center periphery dynamic is not necessarily about physical locations, but eventually about the willingness to imitate or reproduce patterns of domination. Consequently, a global comparative survey may provide a complementary view on the diversity of conditions of research, but also on its systemic inequalities.

These inequalities derived from linguistic, economic and political hegemony are highly visible in our results. There are strong and persistent differences between the different regions as regards the availability of funding, technical infrastructures, and time available for research. Respondents from countries of the Global North, most of which are considered liberal democracies, report more time for research and have higher satisfaction with their facilities and internal funding. Yet, the flourishing of science does not follow strict lines of political geography. Many national states have aligned to global models of scientific knowledge production particular in specific fields of science leading to broad participation of scholars world-wide. As our survey reveals, scholars located in autocratic regimes, particularly in China also enjoy endowment for research as the scholars located in authoritarian states are represented in these fields are also more satisfied with their infrastructure, though external funding is not on the same standard.

These data from perceptions of researchers provide a complementary perspective on bibliometric development. Bibliometric data show that the share of publications from autocratic countries grows considerably fast. Moreover, there is a strong tendency to adapt to existing structures of global science, with a stronger orientation towards international indexation, collaboration, and exchange. The survey data reveal this pattern as particularly respondents from countries categorized as closed autocracies report particularly high internationalization attempts.

As regards the conditions for research, the survey highlights a more complex picture. Scholars from the Global South report less time and resources for conducting research. Instead, they have higher teaching duties, apparently due to set priorities in their local or national contexts. There are apparently also suffering worse conditions regarding technical infrastructures and technical instruments resources provided to the researchers, as respondents from Global South report more dissatisfaction with such conditions compared to their counterpart of the Global North. The actual conditions may be even worse than these reports suggest given that respondents are inclined to provide societal expectable answers. The data also provide more complex patterns as regards the organizational contexts for research with a broad variety of possible end users that may also affect the width of topical choices.

Summarizing, we find that the survey perspective is a valuable resource for further developing the idea of global research cultures, highlighting the epistemic diversity of scholarly contributions and its formats as well as its organizational variety. In opposition to somewhat homogenized picture of what Marginson (2022) describes as global science with specific research type indexed in large databases, output reported here also provides ample evidence of biblio-diversity.

## Statement of ethical approval

It was not possible to obtain approval by an official ethics committee at the time the survey had been conducted. An official ethics committee statute for was established in January 2023. Yet, study design and questionnaire had been checked by all team members for sensitive issues to ensure that the research performed is in accordance with the Declaration of Helsinki. In addition, the questionnaire was checked in advance by the data security officer.

## Statement on informed consent

This research was conducted in accordance with current European data protection legislation (the DSGVO). Written informed consent was obtained from all respondents prior to them answering the survey questions. All respondents were informed of their rights under the current data protection legislation.

## Data Availability

Zenodo. Contemporary global research cultures: results from a global survey about research conditions. https://doi.org/10.5281/zenodo.18197687. ( Lovakov et al., 2026a). This project contains the following underlying data:
• Contemporary global research cultures: results from a global survey about research conditions: This file includes survey data from a global survey on research conditions. Due to ethical concerns, the data set does not include open ended questions from respondents. Data is available under the terms of the Creative Commons Attribution 4.0 International. Zenodo. Appendix for Contemporary global research cultures: results from a global survey about research conditions. https://doi.org/10.5281/zenodo.18197853. ( Lovakov et al., 2026b). This project contains the following underlying data:
• Appendix for Contemporary global research cultures: results from a global survey about research conditions. This file includes figures and tables from the analysis of the survey data. Data is available under the terms of the Creative Commons Attribution 4.0 International. Zenodo. Survey questions of a global survey about research conditions. https://doi.org/10.5281/zenodo.19063125. ( Blümel et al., 2026). This project contains the following underlying data:
• Survey questions from a global survey about research conditions. This file includes all survey questions underlying the analysis of this paper. Data is available under the terms of the Creative Commons Attribution 4.0 International.

## Footnotes

^1^

https://www.researchgate.net/blog/state-of-the-research-community-survey . Accessed 22 June 2025

^2^

https://www.dzhw.eu/en/services/meldungen/detail?pm_id=1590. Accessed 22 June 2025

^3^

https://www.researchgate.net/blog/thank-you-for-participating-in-the-state-of-the-research-community-survey . Accessed 22 June 2025
