# Supplementary material for: Contemporary global research cultures: results from a global survey about research conditions
Source: F1000Res. 2026 Apr 19;15:591. [Version 1] doi: 10.12688/f1000research.171842.1 (PMC13264692; doi:10.12688/f1000research.171842.1)
Supplement: Peer review history

# Reviewer report: Reviewer response for version 1

**DOI:** 10.5256/f1000research.189500.r484618

**Published:** 2026-06-13

**Authors:** Kimkong Heng

**Affiliations:** 1 Pannasastra University of Cambodia (Ringgold ID: 231161), Phnom Penh, Cambodia

Dear Authors,

 Thanks for this research. The data presented are interesting. I have some comments for consideration to improve the quality of the paper.

  

1. Authors should provide reasons why they focus on only three key dimensions of professional environment (p. 5). Also, please cite some references when defining those dimensions.
2. Is there any existing research that explores these dimensions when investigating similar topics? What is the existing research related to each of these dimensions?
3. The four types of regimes mentioned in the methods should be briefly explained.
4. The order of information/findings presented could be revised. For example, Figure 6 about positions could be placed earlier before other analyses. Authors should try to group demographic data together so there is a clear focus on data presentation.
5. The discussion of the findings is relatively limited. Authors should engage relevant research in the interpretation of the findings in the conclusion section. For example, paragraphs 2-4 in the conclusion may be revised and reference to similar prior research should be made.
6. There are a number of tables in the Appendix but I could not see the Appendix and therefore not able to comment on them.

Is the work clearly and accurately presented and does it cite the current literature?

Partly

If applicable, is the statistical analysis and its interpretation appropriate?

I cannot comment. A qualified statistician is required.

Are all the source data underlying the results available to ensure full reproducibility?

Yes

Is the study design appropriate and is the work technically sound?

Yes

Are the conclusions drawn adequately supported by the results?

Yes

Are sufficient details of methods and analysis provided to allow replication by others?

Yes

Reviewer Expertise:

Higher education research

I confirm that I have read this submission and believe that I have an appropriate level of expertise to confirm that it is of an acceptable scientific standard.

# Reviewer report: Reviewer response for version 1

**DOI:** 10.5256/f1000research.189500.r479498

**Published:** 2026-04-30

**Authors:** Mohd Mokhtar Muhamad

**Affiliations:** 1 University Putra Malaysia, Serdang, Selangor, Malaysia

Insightful research that relates research conditions with different political regime. While the author adopts the V-Dem classification to provide methodological rigor, relying on the term 'authoritarian' risks reinforcing a center-periphery hierarchy that views non-Western scientific growth as a political anomaly rather than a legitimate expression of 'epistemic diversity.' Nevertheless, the authors did report that political regime type is an insufficient proxy for scientific health.

Is the work clearly and accurately presented and does it cite the current literature?

Yes

If applicable, is the statistical analysis and its interpretation appropriate?

I cannot comment. A qualified statistician is required.

Are all the source data underlying the results available to ensure full reproducibility?

Yes

Is the study design appropriate and is the work technically sound?

Yes

Are the conclusions drawn adequately supported by the results?

Yes

Are sufficient details of methods and analysis provided to allow replication by others?

Yes

Reviewer Expertise:

philosophy of education

I confirm that I have read this submission and believe that I have an appropriate level of expertise to confirm that it is of an acceptable scientific standard.
